# The Joint Associations of Physical Activity and Sedentary Behaviors on Adiposity during Adolescence: The 1993 Pelotas (Brazil) Cohort Study

**DOI:** 10.3390/children10020265

**Published:** 2023-01-31

**Authors:** Soyang Kwon, Fernando C. Wehrmeister, Helen Gonçalves, Bruna Gonçalves C. da Silva, Ana M. B. Menezes

**Affiliations:** 1 Department of Pediatrics, Northwestern University, Chicago, IL 60611, USA; 2 Post-Graduate Program in Epidemiology, Federal University of Pelotas, Peloas CEP96020-220, RS, Brazil

**Keywords:** children, cohort study, sedentary time, television (TV) viewing, ActiGraph accelerometers, dual-energy X-ray absorptiometry (DXA)

## Abstract

A prior study conducted in high-income countries demonstrated that specific sedentary behavior, such as TV viewing, is prospectively associated with adiposity in both active and inactive adolescents. The aim of this study was to examine the joint associations of sedentary behaviors and moderate- and vigorous-intensity physical activity (MVPA) with adiposity among Brazilian adolescents. This prospective cohort study included 377 participants of the 1993 Pelotas (Brazil) Study who completed an accelerometry assessment at age 13 years and a dual-energy X-ray absorptiometry (DXA) assessment at age 18 years. Accelerometer-measured MVPA was dichotomized into high (≥60 min/day) and low (<60 min/day). Accelerometer-measured sedentary time (SED) was dichotomized into low (<49 min/h) and high (≥49 min/h) based on the median. Self-reported TV viewing time was also dichotomized into low (<3 h/day) and high (≥3 h/day) based on the median. We combined the two MVPA groups (high and low) and two SED groups (low and high) to form the four MVPA&SED groups: high&low, high&high, low&low, and low&high. We also created four MVPA&TV groups in the same manner. Fat mass index (FMI; kg/m^2^) was calculated using DXA-derived fat mass. Multivariable linear regression analyses compared FMI at 18 years among the four MVPA&SED groups and among the four MVPA&TV groups, adjusting for socioeconomic status, energy intake, and baseline adiposity. The analysis results showed that SED or TV viewing time was not prospectively associated with adiposity in both active and inactive Brazilian adolescents. This study suggests that the association between specific sedentary behaviors, such as TV viewing, and adiposity may differ across societal settings—in this case, high-income vs. middle-income countries.

## 1. Introduction

Physical activity (PA) is well recognized to provide numerous health benefits in youth, including obesity prevention, cardiovascular health, and bone health [1]. Sedentary behaviors have also gained great attention as a potential independent risk factor for these health outcomes [2,3,4,5]. However, supporting evidence for this premise is limited for youth. Scientific reviews for the 2018 United States (US) Physical Activity Guidelines (PAG) [1] and the 2020 World Health Organization (WHO) PAG [6] identified a knowledge gap regarding the effects of sedentary behaviors on health outcomes, such as obesity, in youth. The US PAG review [1] particularly highlighted the lack of knowledge on the interactive effects of physical activity and sedentary behaviors on health outcomes in youth. In adults, studies [7,8,9,10] have shown that the relationship between sedentary behavior and these health outcomes varies by moderate- and vigorous-intensity PA (MVPA) levels, in which the harmful effects of sedentary behaviors is attenuated by high levels of physical activity. Therefore, it is plausible that the effects of sedentary behaviors on health outcomes among youth could differ by their physical activity levels. 

Griffiths et al. [11] examined the prospective joint associations of MVPA and total sedentary time (SED) at age 7 years with adiposity at age 11 years in a nationally representative sample of 6497 British children participating in the Millennium cohort study and found that SED was not associated with adiposity, regardless of the levels of MVPA. Our prior study [2] also examined the prospective joint associations of MVPA and sedentary behaviors with adiposity in 2619 British adolescents participating in the Avon Longitudinal Study of Parents and Children (ALSPAC). In that study, we found no association between SED during adolescence (age 11 to 15 years) and adiposity at age 17 years in both active (MVPA ≥ 60 min/day) and inactive (MVPA < 60 min/day) British adolescents; however, we found a positive association between television (TV) viewing time and adiposity in both active (MVPA ≥ 60 min/day) and inactive (MVPA < 60 min/day) UK adolescents [2]. TV viewing time is a specific type of sedentary behavior that has often been reported to be positively associated with adiposity in youth [1]. 

In low- and middle-income countries (LMIC), studies about the effects of sedentary behavior on adiposity are scarce [12], although obesity is a global health risk shared by low-, middle-, and high-income countries [13]. In middle-income counties, such as Brazil, the associations between sedentary behavior and adiposity could differ from those in high-income countries, given that the associations of sedentary time and adiposity with societal factors (e.g., socioeconomic status) have been reported to differ between the two settings: inverse associations in high-income countries versus positive or no associations in middle-income countries [14,15,16]. 

The purpose of this study was to prospectively examine the joint associations of MVPA and SED or TV viewing time with adiposity development among Brazilian adolescents. Compared to prior prospective cohort studies that examined the association between sedentary behavior and adiposity in youth, this investigation is unique in that it was designed to examine the association separately for adolescents who are active and those who are not, it used dual-energy X-ray absorptiometry (DXA)-derived fat mass as an adiposity indicator (as opposed to body mass index [BMI]), and its sample was from a middle-income country.

## 2. Materials and Methods

### 2.1. Study Participants

This study used data obtained from the 1993 Pelotas (Brazil) cohort study. The 1993 Pelotas Study is a birth cohort study that recruited almost all new births (n = 5249) born from all maternity hospitals in the city of Pelotas, Brazil, in 1993 [17]. The City of Pelotas is located in southern Brazil with over 300,000 inhabitants, with markedly high socioeconomic inequalities [17]. The 1993 Pelotas Study sample does not represent all Brazilian births. In 2006 and 2007, 568 cohort members who had completed all previous assessments were sought and 511 adolescents were located and interviewed for 13-year assessments. A comparison of the characteristics between the entire cohort and the subsample can be found in a previous publication [18]. Later, the entire cohort was invited to 18-year assessments. 

For the current paper, we included 1993 Pelotas Study participants who completed an accelerometer assessment at age 13 years (the primary exposure) and a dual-energy X-ray absorptiometry (DXA) examination at age 18 years (the primary outcome). We excluded those were classified as obese (WHO BMI-for-age z-score ≥ 2.00) [19] at age 13 years. The study protocol of the Pelotas Study was approved by the Federal University of Pelotas Institutional Review Boards (IRB). At age 17 years or younger, written informed consent was obtained from parents or guardians and assent was obtained from participants. At age 18 years or older, written informed consent was obtained from participants.

### 2.2. Measurements

Exposures: The main exposure variables were time spent in MVPA, SED, and TV viewing during adolescence. During each of the 11-, 13-, and 15-year study visits, participants self-reported TV viewing time in minutes on a weekday and a weekend day in face-to-face interviews [20]. Daily TV viewing time (h/day) was calculated using the following equation: (TV viewing time in hours on a weekday × 5 + TV viewing time in hours on a weekend day × 2) ÷ 7. TV viewing time during adolescence was estimated by averaging daily TV viewing time across the three time points. This TV viewing assessment method has been used in prior publications [16,20]. 

MVPA and SED were assessed using accelerometers. Accelerometers are widely used to assess child PA [21]. Accelerometer assessment was conducted at age 13 years but not at age 11 or 15 years. During the 13-year study visit, participants were asked to wear an ActiGraph GT1M accelerometer on the hip for 4 consecutive days and 24 h/day, typically from Thursday to Sunday [17]. After excluding non-wear data [17] as defined by the Choi algorithm [22], we identified participants with ≥3 valid monitor wear days (defined as ≥600 min/day) to include in the data analysis. Daily minutes spent in MVPA (defined as ≥2000 counts per minute) and hourly minutes spent in sedentary behavior (defined as <100 counts per minute) were calculated [17].

Outcome: The primary outcome variable was the fat mass index (FMI) derived from DXA scans at age 18 years. DXA is a gold standard for fat mass assessment. During the 18-year study visit, participants were asked to participate in a DXA scan (GE Lunar Prodigy, GE Healthcare, Boston, MA, USA) [20]. Participants were ineligible for the DXA scan if they used a wheelchair or had osteoarticular deformities, implanted metal pins, screws, plates and non-removable metallic objects (e.g., body piercings and/or chains), or were extremely obese, had a height over 1.92 m, or pregnant [23]. The DXA scan was performed with participants in supine position. All procedures were conducted following the best practice by trained staff. More detailed DXA procedures are available in a prior publication [24]. Scan images were analyzed using the in-built GE Lunar enCore software, which produced fat mass estimation. Standing height was measured using a stadiometer with 0.1 cm precision. FMI was calculated by dividing fat mass (kg) by height squared (m^2^). 

Confounding factors: Consistent with our prior study [2], we considered the followings as confounding factors: sex, BMI z-score at the 13-year assessment, and age, wealth index quintiles, and energy intake quintiles at the 18-year assessment. Wealth index, a relative wealth indicator within a county or population, is commonly used to measure socioeconomic position [25]. The wealth index considers household construction materials, water and sanitation access, and ownership of various assets (e.g., TV) to assess the relative wealth within a country or population. The wealth index was divided into quintiles [26]. Daily energy intake (kcal/day) was estimated using a food frequency questionnaire [27] and categorized into five quintile groups based on sex-specific quintile cut-points. 

### 2.3. Statistical Analysis

All analyses were conducted separately according to participant sex, using SAS 9.4 (Cary, CA, USA). Missing data were treated using a list-wise deletion method. MVPA at age 13 years was dichotomized into high (≥60 min/day) and low (<60 min/day) [6]. SED at age 13 years was dichotomized into low (<49 min/h) and high (≥49 min/h) based on the median. Average TV viewing measured at ages 11, 13, and 15 years was also dichotomized into low (<3 h/day) and high (≥3 h/day) based on the median. We used averaged TV viewing data measured at three time-points to better reflect TV viewing behavior throughout the adolescent period. Because MVPA was measured only at one time-point at age 13 years, it was inappropriate to conduct longitudinal data analysis (e.g., trajectory analysis, mixed model analysis) to examine the joint MVPA and TV viewing variable. We combined the two MVPA groups (high and low) and the two SED groups (low and high) to form four MVPA&SED groups: high&low, high&high, low&low, and low&high. We also created four MVPA&TV groups in the same manner. 

Descriptive analyses, including frequency and distribution analyses, were conducted. We calculated the means of the main study variables according to wealth index quintiles. Multivariable linear regression analyses were conducted with a log link to compare FMI at 18 years among the four MVPA&SED groups, adjusted for BMI z-score at the 13-year assessment, and age, wealth index quintiles, and energy intake quintiles at the 18-year assessment. The regression analyses were repeated for the MVPA&TV groups. A significance level was set at 0.05 (two-sided). 

## 3. Results

**Figure 1:**
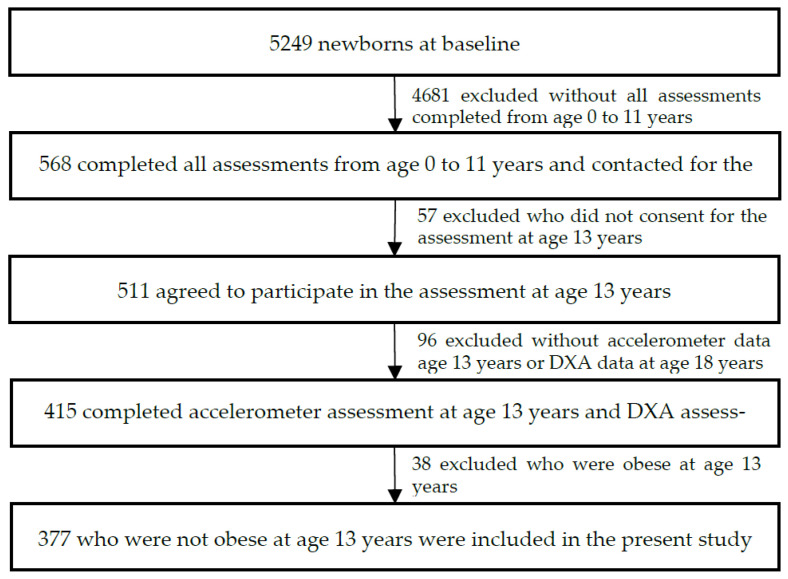
Flow chart depicting the 1993 Pelotas Study participants included in the present study.

Of the 415 Pelotas Study participants who had accelerometry and DXA data, we excluded 38 participants (9.2%) with obesity at the 13-year assessment. A total of 377 Pelotas participants (189 males and 188 females) were included for analysis. Figure 1 illustrates the participant flow chart.

Table 1 presents the means of the primary exposure and outcome variables according to wealth index quintiles among males. In both males and females, when participants were from a wealthier family, MVPA levels were lower (trend *p* < 0.05): 86 vs. 59 min/day for the lowest vs. highest wealth index quintile groups among males and 60 vs. 50 min/day for the lowest vs. highest wealth index quintile groups among females. In contrast, when participants were from a wealthier family, SED levels tended to be higher; however, the trends were not statistically significant. 

Average TV viewing time during adolescence tended to increase with higher wealth index among males, while it tended to decrease with higher wealth index among females (Table 2); however, the trends were not statistically significant. Among males, FMI tended to be higher with a higher wealth index. Among females, FMI tended to be lower in the highest and the lowest wealth index quintiles, compared to the other three quintile groups. 

Males (Table 3): When unadjusted means were compared by the four MVPA&SED groups, the high MVPA group (unadjusted mean FMI = 3.2; 95% CI = 2.8, 3.7) tended to have a lower FMI compared to the low MVPA (unadjusted mean FMI = 4.0; 95% CI = 2.4, 5.6) within the low SED group. Similarly, the high MVPA group (unadjusted mean FMI = 3.5; 95% CI = 2.4, 4.6) tended to have a lower FMI compared to the low MVPA (unadjusted mean FMI = 4.0; 95% CI = 3.2, 4.7) within the high SED group. When unadjusted means were compared between the four MVPA&TV groups, the high MVPA group (unadjusted mean FMI = 3.1; 95% CI = 2.5, 3.7) tended to have a lower FMI, compared to the low MVPA (unadjusted mean FMI = 3.9; 95% CI = 2.8, 5.0) within the low TV group. Similarly, the high MVPA group (unadjusted mean FMI = 3.4; 95% CI = 2.9, 3.9) tended to have a lower FMI compared to the low MVPA (unadjusted mean FMI = 4.0; 95% CI = 3.2, 4.9) within the high TV group. However, there was no significant difference in FMI between the low and high SED groups and between the low and high TV viewing groups within each of the high and low MVPA groups.

When FMI was adjusted for the confounding factors, it was significantly higher in the low MVPA&high TV group (adjusted mean FMI = 4.0; 95% confidence interval [CI] = 3.2, 4.9) compared to the high MVPA&low TV group (adjusted mean FMI = 3.1; 95% CI = 2.7, 3.5). However, no significant differences in the FMI were found among the four MVPA&SED groups.

Females (Table 4): The higher TV viewing group tended to have a higher FMI within each of the high MVPA (FMI = 8.3 kg/m^2^ for high TV viewing vs. 7.9 kg/m^2^ for low TV viewing) and low MVPA (FMI = 8.4 kg/m^2^ for high TV viewing vs. 7.9 kg/m^2^ for low TV viewing) groups; however, the differences did not reach a statistical significance. When FMI was adjusted for the confounding factors, no significant FMI differences were found among the MVPA&SED groups or among the MVPA&TV groups. 

## 4. Discussion

This study found that total sedentary time or TV viewing time was not prospectively associated with adiposity during adolescence in both active and inactive Brazilian adolescents. The present study also found that the relationships between socioeconomic status and MVPA, sedentary time, TV viewing, and adiposity in Brazilian adolescents are not linear, and are more complex than the relationships observed in high-income countries: i.e., inverse associations between socioeconomic status and unhealthy PA, sedentary time, and adiposity [14]. 

This study found that, relative to adolescents in high-income countries, such as the UK and US [2,28,29], Brazilian adolescents in the current Pelotas Study sample were active: 73 min/day and 56 min/day of MVPA on average for males and females aged 13 years, respectively. However, Brazilian adolescents, both males and females, tended to engage in lower PA, as they are of higher socioeconomic status. Although we were unable to examine domain-specific PA with accelerometer data, it is feasible that, as socioeconomic status is higher in a middle-income country, transportation PA in a daily life may be lowered with an increase in car use. Unlike PA behaviors, the variations in total sedentary time and TV viewing time were relatively small across the wealth index quintiles, showing no clear trend in the relationship between socioeconomic status and sedentary behaviors. The relationship between socioeconomic status and adiposity appeared to differ by sex: males in the highest wealth index quintile had the highest FMI, while females in the highest and lowest health index quintiles had the lowest FMI. All these findings represent complex relationships between socioeconomic status and adiposity or obesogenic behaviors in middle-income counties, which are different from those in high-income countries. 

This study found no prospective association between TV viewing and adiposity among Brazilian adolescents, which contradicts that which has been hypothesized and supported based on data from high-income counties [2,30,31]. For example, our prior study [21] found from a UK sample that higher TV viewing time at ages 13 and 16 years was prospectively positively associated with adiposity at age 17 years in both active and inactive British adolescents. However, the findings of the present study are consistent with an investigation by Mielke et al. [20] that used self-reported sedentary time data in the entire 1993 Pelotas Study sample (n = 3613). We speculate that the pathway linking TV viewing and adiposity could be modified by societal factors (i.e., socioeconomic status and food security) among Brazilian adolescents. For example, the findings could be partly due to the nutrition transition [14]. That is, in middle-income countries, those with higher socioeconomic status have greater food security and access to energy dense food; as countries develop, food, particularly energy dense food, becomes more abundant and accessible, even to those of lower socioeconomic status. Therefore, it is plausible that, in middle-income countries, energy dense snacks may not be readily accessible while watching TV, which could modify the link between TV viewing and adiposity. Inconsistent and complex associations between socioeconomic status and adiposity among Brazilians [32], which are modified by sex, adds to the challenge of understanding the contributions of socioeconomic status and TV viewing behaviors to obesity or weight gain. To account for the influences of socioeconomic status and energy intake, the current study controlled statistically for the effects of the wealth index quintile and energy intake quintile variables. However, given the complex relationships among socioeconomic status, energy intake, TV viewing, and adiposity, our results may have been distorted by potential residual confounding [33] due to the classification errors of these variables and additional confounding factors that were not considered. 

The practical implications of this study include the fact that the potential harmful effects of TV viewing on excess body fat should not be generalized across settings, particularly in middle-income countries. While the current PAG by the WHO [6] should guide PA recommendations in the middle-income countries, such as Brazil, establishing a country-specific PAG might be necessary. 

The strengths of this study include the use of DXA-derived fat mass as an adiposity indicator and the use of a middle-income country as a study setting. A limitation of this study is its relatively small sample size for the examination of the joint associations. Moreover, a single assessment of MVPA and total sedentary time at age 13 years may not represent MVPA and the total sedentary time during the entire adolescent period. Self-reported TV viewing time is subjective and prone to error. The results could have been biased due to unmeasured or poorly measured confounding factors. The results may not be generalized to other adolescent populations from different settings.

Future research is guaranteed to gather empirical evidence for the roles of sedentary behaviors and socioeconomic status in adiposity in low- and middle-income countries, as the present study suggests that relationships between socioeconomic status and adiposity or obesogenic behaviors appear to be different in middle-income countries from those in high-income countries. Moreover, further research should identify specific types of sedentary behaviors (e.g., TV viewing, reading) that influence adiposity. 

## 5. Conclusions

This study found that total sedentary time or TV viewing time was not prospectively associated with adiposity in both active and inactive Brazilian adolescents. This study suggests that the association between specific sedentary behaviors, such as TV viewing, and adiposity, may differ across societal settings, in this case, in high-income vs. middle-income countries.

## Figures and Tables

**Table 1:** Means of MVPA, sedentary time, TV viewing, and FMI according to wealth index quintiles among males.

Wealth Index, Quintiles	Sample Size	MVPA at Age 13, min/Day	Sedentary Time at Age 13, min/h	Average TV Viewing at Age 11, 13, and 15, h/Day	FMI at Age 18, kg/m^2^
		Mean ± SD	Mean ± SD	Mean ± SD	Mean ± SD
Males	189	73 ± 27	47 ± 3	3.1 ± 1.5	3.5 ± 2.4
1 (lowest)	38	86 ± 26	45 ± 3	2.8 ± 1.6	3.2 ± 2.8
2	35	79 ± 32	47 ± 3	3.1 ± 1.2	2.6 ± 1.6
3	51	69 ± 24	48 ± 3	3.2 ± 1.7	3.9 ± 2.4
4	28	71 ± 26	48 ± 3	3.0 ± 1.4	3.7 ± 2.2
5 (highest)	37	59 ± 22	49 ± 2	3.1 ± 1.3	4.1 ± 2.3

* Footnotes: FMI, fat mass index; MVPA, moderate- and vigorous-intensity physical activity; TV, television viewing; SD, standard deviation.

**Table 2:** Means of MVPA, sedentary time, TV viewing, and FMI according to wealth index quintiles among females.

Wealth Index, Quintiles	Sample Size	MVPA at Age 13, min/Day	Sedentary Time at Age 13, min/h	Average TV Viewing at Age 11, 13, and 15, h/Day	FMI at Age 18, kg/m^2^
		Mean ± SD	Mean ± SD	Mean ± SD	Mean ± SD
Females	188	56 ± 23	49 ± 3	3.4 ± 1.6	8.1 ± 3.2
1 (lowest)	33	60 ± 23	49 ± 2	3.5 ± 1.4	7.4 ± 2.3
2	30	59 ± 31	49 ± 3	3.8 ± 1.6	8.2 ± 3.4
3	53	57 ± 21	49 ± 3	3.4 ± 1.5	8.7 ± 3.7
4	37	54 ± 21	50 ± 3	3.6 ± 1.7	8.5 ± 3.1
5 (highest)	35	50 ± 18	51 ± 2	2.9 ± 1.5	7.6 ± 2.9

* Footnotes: FMI, fat mass index; MVPA, moderate- and vigorous-intensity physical activity; TV, television viewing; SD, standard deviation.

**Table 3:** Unadjusted and adjusted means of fat mass index among MVPA&SED and MVPA&TV groups among males.

	Sample Size	Unadjusted Mean (95% CI)	Adjusted Mean (95% CI)
MVPA&SED group			
High&low	107	3.2 (2.8, 3.7)	3.1 (2.7, 3.5)
High&high	15	3.5 (2.4, 4.6)	3.4 (2.6, 4.4)
Low&low	19	4.0 (2.4, 5.6)	3.6 (2.9, 4.5)
Low&high	48	4.0 (3.2, 4.7)	3.3 (2.8, 3.9)
*p*-value for the group variable		0.28	0.53
MVPA&TV group			
High&low	60	3.1 (2.5, 3.7)	3.1 (2.7, 3.5)
High&high	62	3.4 (2.9, 3.9)	3.1 (2.7, 3.5)
Low&low	28	3.9 (2.8, 5.0)	3.3 (2.7, 3.9)
Low&high	39	4.0 (3.2, 4.9)	3.8 (3.2, 4.4)
*p*-value for the group variable		0.23	0.53

* Footnotes: CI, confidence interval; MVPA, moderate- and vigorous-intensity physical activity; TV, television viewing; SED, total sedentary time. Adjusted means were calculated, adjusting for BMI z-score at the 13-year assessment, and age, wealth index quintiles, and energy intake quintiles at the 18-year assessment.

**Table 4:** Unadjusted and adjusted means of fat mass index among MVPA&SED and MVPA&TV groups among females.

	Sample Size	Unadjusted Mean (95% CI)	Adjusted Mean (95% CI)
MVPA&SED group			
High&low	59	8.3 (7.4, 9.2)	7.8 (7.3, 8.4)
High&high	13	7.4 (6.0, 8.8)	8.0 (6.8, 9.4)
Low&low	24	7.5 (6.2, 8.6)	7.8 (7.0, 8.8)
Low&high	92	8.3 (7.7, 9.0)	7.6 (7.1, 8.1)
*p*-value for the group variable		0.54	0.94
MVPA&TV group			
High&low	27	7.8 (6.9, 8.9)	7.9 (7.1, 8.8)
High&high	45	8.3 (7.2, 9.4)	7.8 (7.2, 8.5)
Low&low	45	7.9 (7.0, 8.7)	7.9 (7.3, 8.6)
Low&high	71	8.4 (7.6, 9.2)	7.7 (7.2, 8.2)
*p*-value for the group variable		0.88	0.94

* Footnotes: CI, confidence interval; MVPA, moderate- and vigorous-intensity physical activity; TV, television viewing; SED, total sedentary time. Adjusted means were calculated, adjusting for BMI z-score at the 13-year assessment, and age, wealth index quintiles, and energy intake quintiles at the 18-year assessment.

## Data Availability

Data may be available upon request to Ana MB Menezes (anamene.epi@gmail.com) at Federal University of Pelotas, Brazil.
